# Discrimination Task Reveals Differences in Neural Bases of Tinnitus and Hearing Impairment

**DOI:** 10.1371/journal.pone.0026639

**Published:** 2011-10-31

**Authors:** Fatima T. Husain, Nathan M. Pajor, Jason F. Smith, H. Jeff Kim, Susan Rudy, Christopher Zalewski, Carmen Brewer, Barry Horwitz

**Affiliations:** 1 Department of Speech and Hearing Science, University of Illinois at Urbana-Champaign, Champaign, Illinois, United States of America; 2 Brain Imaging and Modeling Section, National Institute on Deafness and Other Communication Disorders, National Institutes of Health, Bethesda, Maryland, United States of America; 3 Otolaryngology Branch, National Institute on Deafness and Other Communication Disorders, National Institutes of Health, Bethesda, Maryland, United States of America; The University of Hong Kong, Hong Kong

## Abstract

We investigated auditory perception and cognitive processing in individuals with chronic tinnitus or hearing loss using functional magnetic resonance imaging (fMRI). Our participants belonged to one of three groups: bilateral hearing loss and tinnitus (TIN), bilateral hearing loss without tinnitus (HL), and normal hearing without tinnitus (NH). We employed pure tones and frequency-modulated sweeps as stimuli in two tasks: passive listening and active discrimination. All subjects had normal hearing through 2 kHz and all stimuli were low-pass filtered at 2 kHz so that all participants could hear them equally well. Performance was similar among all three groups for the discrimination task. In all participants, a distributed set of brain regions including the primary and non-primary auditory cortices showed greater response for both tasks compared to rest. Comparing the groups directly, we found decreased activation in the parietal and frontal lobes in the participants with tinnitus compared to the HL group and decreased response in the frontal lobes relative to the NH group. Additionally, the HL subjects exhibited increased response in the anterior cingulate relative to the NH group. Our results suggest that a differential engagement of a putative auditory attention and short-term memory network, comprising regions in the frontal, parietal and temporal cortices and the anterior cingulate, may represent a key difference in the neural bases of chronic tinnitus accompanied by hearing loss relative to hearing loss alone.

## Introduction

Subjective tinnitus is the phantom perception of sound in the absence of an external source. The annoyance and distress associated with tinnitus range from mild to severe, with the latter type having a major impact on a person's life, making sleep difficult and intellectual work challenging [1]. The incidence of tinnitus is higher above the age of 50, consistent with the increased incidence of hearing loss with age. As per the National Center for Health Statistics survey (1999) of non-institutionalized Americans reported in [2], approximately 200 men and 100 women per 1000 persons suffer from hearing loss in the 45–64 year range and approximately 70 men and 40 women per 1000 persons suffer from tinnitus in the same 45–64 year age range. Hearing loss causes reorganization of the central auditory processing pathways and associated areas in the brain, possibly leading to tinnitus. However, not everyone with hearing loss has tinnitus and about 10% of those with tinnitus have normal hearing [2], [3]. One of the challenges in studying the neural bases of chronic tinnitus is dissociating the effects and mechanisms of tinnitus from those of hearing loss alone. The nature of the interaction between hearing loss and tinnitus has long been noted. Hearing loss is the most common risk factor for developing tinnitus and the most correlated condition with tinnitus [1], [4], [5]. Few studies, however, have taken hearing loss into account when investigating the neural correlates of tinnitus. Most studies have either compared patients with tinnitus and hearing loss with normal hearing controls [6], compared participants with normal hearing and tinnitus with normal hearing controls [7], [8], [9], or used specific paradigms that allowed participants to serve as their own controls [10], [11], [12]. In the present study, we chose to use two control groups against which to compare the group with tinnitus and hearing loss: those with normal hearing and those with similar hearing loss. The inherent assumption was that comparing participants with tinnitus and hearing loss against participants with hearing loss alone would allow us to better identify the neural correlates of tinnitus. This, of course, may be a simplification in that it is possible that the underlying pathophysiology of tinnitus with hearing loss differs from that with tinnitus and normal hearing. Increasing evidence from brain imaging studies suggests that large-scale networks subserving attention, emotion, and cognition are affected in chronic tinnitus [10], [11], [12], [13], [14], [15]. However, the engagement of these networks has been implicated indirectly rather than through explicit tasks targeting attention, cognition or emotional processing. At the same time, animal studies have noted the involvement of multimodal networks in tinnitus [16], [17]. The current study was therefore designed to identify differences in the sensory and cognitive networks across chronic tinnitus and hearing loss conditions, possibly related to short-term memory and auditory processing. Short-term memory tasks have been successfully used to investigate influences of aging and hearing loss on cognitive behavior [18], [19], [20], [21], [22]. Depending on the task being used, brain imaging studies have investigated neural bases of attention, working memory, and cognitive and sensory effort in older adults with normal hearing or hearing loss. Our study was motivated in part by recent behavioral studies [23], [24], [25] that corroborate anecdotal evidence of the distracting effect of tinnitus on real-world tasks involving sounds. In the behavioral studies, deficits ascribed to tinnitus in cognitive and other demanding tasks typically take the form of slower responses rather than lower accuracy. This would suggest that subjects with tinnitus in our study would exhibit longer reaction time than those without tinnitus in completing an active discrimination task. Further, in the present study we account for hearing loss that often accompanies tinnitus and may contribute to the severity and annoyance of tinnitus [1], [26] and may result in structural changes [27], [28].

We used two tasks to investigate the neural bases of short-term memory and auditory processing in individuals with hearing loss without tinnitus, participants with hearing loss accompanied by tinnitus and normal hearing control subjects without tinnitus. Both tasks used identical non-speech stimuli; they differed only in the tasks: passive listening or active discrimination. We had previously used similar tasks and stimuli to investigate short-term memory and auditory processing in young normal-hearing adults [29]. The stimuli included pure tones and frequency-modulated sweeps that were employed in separate scanning (echo-planar imaging) runs [29]. Relative to rest, a distributed network involving the temporal, frontal and parietal cortices was preferentially activated for the discrimination task. In a recent pitch discrimination fMRI study of individuals with tinnitus [30], participants discriminated a series of tonal pips with three different frequencies. Activations in the middle frontal gyrus, putamen and left-hemispheric insula were observed in six tinnitus patients and in the right hemispheric anterior insula for the six controls in addition to auditory cortices, for the task relative to the rest. They also found the caudate nucleus, superior frontal gyrus and anterior cingulate to be more responsive in the tinnitus patients compared to the controls. The two studies [29], [30] suggest that discrimination task using simple non-speech sounds is a valid method to differentiate some of the neural correlates of tinnitus from those of normal hearing or hearing loss. However, note that in the Wunderlich study, the participants in the tinnitus group had various degrees of hearing loss, ranging from none to mild, especially at higher frequencies. The small number of subjects and the lack of controls with hearing loss limit the usefulness of the study.

We wanted to test differences in behavior and neural response of those with hearing loss with and without tinnitus, for sounds they could hear and discriminate well. Both of these groups would be compared to a control group of normal hearing participants without tinnitus. We ensured that all participants could hear the sounds equally well by (a) recruiting only those with either normal hearing for octave frequencies 0.25–8 kHz (control group) or normal hearing through 2 kHz (i.e. they have bilateral high-frequency sensorineural hearing loss) and (b) creating stimuli that included frequencies only up to 2 kHz. Our prediction was that the response of a distributed set of regions in the frontal, parietal and temporal cortices would be enhanced for those with hearing loss and would be increased further for those with hearing loss and tinnitus compared to normal hearing controls during these tasks. Such increased response would be more apparent for the discrimination task relative to the passive listening task because of the greater engagement of a distributed cortical network in the former, possibly due to attentional and short-term memory processing. The prediction was based on our previous fMRI study of young normal hearing adults using similar stimuli and a discrimination task. However, because all participants could hear the sounds and if their behavior did not differ significantly, the null hypothesis would be that there would be no appreciable difference in the response of the auditory processing network between the normal hearing and hearing impaired group without tinnitus; the group with tinnitus would differ due to the additional distracting factor of chronic tinnitus.

## Methods

### Subjects

Three groups of participants were recruited in the study from the greater Washington, D.C. metropolitan area. All participants gave written informed consent. The National Institutes of Health/National Institute of Neurological Disorders and Stroke-National Institute on Deafness and Other Communication Disorders Institutional Review Board approved the study (protocol 06-DC-0218) and all participants were suitably compensated.

The tinnitus group (TIN) consisted of 8 male volunteers (age range = 42–64 yr, mean = 56.13 yr, SD = 7.04 yr) with bilateral, mild to moderately-severe high-frequency sensorineural hearing loss and chronic subjective tinnitus that had persisted for between 3–38 years at the time of their scan (Table 1). The tinnitus percept was most frequently described as a buzzing, ringing, hissing or a whistle sound. Others described a hum, clear tone or pulsating percept (all subjects denied changes in time with heartbeat or respiration). One subject perceived the sound of cicadas. Five subjects described more than one of the above sounds. Tinnitus severity was evaluated by the Tinnitus Handicap Inventory and all subjects were either grade 1 – slight or grade 2 – mild (range = 10–26, mean = 17.25, SD = 5.01) [31], [32]. We assessed laterality of the tinnitus percept via questionnaires. We excluded potential participants if they did not have symmetrical (bilateral) hearing loss or non-lateralized tinnitus percept. Six subjects experienced their tinnitus bilaterally or in the “middle of the head.” Two subjects described their tinnitus as more left lateralized but still central. Of the 28 (6 female) individuals with tinnitus screened for this study, only 8 male patients met our criteria for symmetrical high-frequency sensorineural hearing loss and chronic tinnitus. The others were not included in the study due to our stringent exclusionary criteria of the type of hearing loss, type of tinnitus, and other physical or mental health issues.

**Table 1:** Demographic and clinical characteristics of the participants in the study.

Variables	Normal HearingN = 11	Hearing LossN = 7	TinnitusN = 8
Age (M/SD)	48.09/10.42	51.38/11.45	56.13/7.04
Sex N (M/F)	11/0	7/0	8/0
BDI-II (M/SD)	0.75/2.81	0.57/0.73	1.45/1.49
THI (M/SD)	n/a	n/a	17.25/5.01
Duration of tinnitus (M/SD) in years	n/a	n/a	14.43/12.56

BDI = Beck Depression Inventory, THI = Tinnitus Handicap Inventory.

The second group (HL) (n = 7) was matched in age (age range = 31–64 yr, mean = 51.38 yr, SD = 11.45 yr), gender and hearing loss and had bilateral, mild to moderately-severe hearing loss but did not have tinnitus. The third group (NH) (n = 11) was age (age range = 32–63 yr, mean = 48.09 yr, SD = 10.42 yr) and gender-matched and had normal hearing with no tinnitus. All subjects scored in the minimal depression range on the Beck Depression Inventory (BDI-II) [33], [34] (range = 0–10, mean for TIN = 1.45, mean for HL = 0.57, mean for NH = 0.75) (Table 1). After initial screening, all potential participants were evaluated by a licensed medical practitioner and were excluded if they had current, or a history of, temporomandibular joint problems, hyperacusis, Meniere's disease, benign positional vertigo or any other health issues that may have presented complications or contraindications with MRI. We explicitly excluded for hyperacusis not only because of possible noise-exposure in the MRI scanner but also because of studies showing elevated auditory activity due to this factor [35].

### Audiometric evaluation

**Figure 1:**
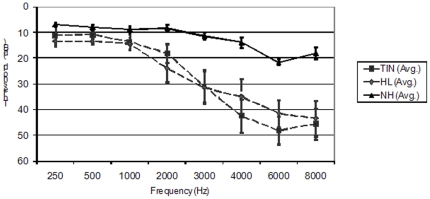
Average audiograms of the participants including error bars that depict standard error of the mean.

All participants underwent full audiologic evaluation before and after the scanning session at the NIH Clinical Center. The audiologic examination, including speech recognition and pure-tone air- and bone-conduction thresholds (0.25–8 kHz), was conducted in a double-walled audiometric test suite using ER-3A transducers in accordance with American National Standards Institute standards (American National Standards Institute, S3.1-1999 American National Standard Maximum Permissible Ambient Noise Levels for Audiometric Test Rooms (Standard S3.1), New York, NY: American National Standards Institute, 2003, and S3.1-1996 American National Standard Specification for Audiometers (Standard S3.6). New York, NY: American National Standards Institute; 2004). Additional audiometric measures, including distortion product otoacoustic emissions, tympanometry, and acoustic reflex thresholds and decay, were conducted to ensure that there were no audiometric signs of conductive or retrocochlear pathology. Loudness tolerance evaluation using recorded samples of scanner noise was also conducted to ensure that each participant's loudness discomfort levels were sufficiently high to permit scanning without loudness discomfort. We excluded potential participants who exhibited symptoms of hyperacusis, either via loudness tolerance evaluation or subjective questionnaire. All participants in the NH group had pure-tone thresholds of 25 dB HL or less for all of the test frequencies. Participants in the TIN and HL groups had pure-tone thresholds of 25 dB HL or less for 0.25–2 kHz, and sensorineural hearing loss in the mild to moderately-severe range (no greater than 70 dB as defined by [36] and noted at http://www.asha.org/public/hearing/disorders/types.htm) for 3–8 kHz. There were no statistically significant differences in the pure-tone average hearing loss (across all testing frequencies) (p = 0.89 using Wilcoxon Rank Sum test) or at the higher frequencies (4, 6, 8 kHz) (p = 0.69 using Wilcoxon Rank Sum test) for the TIN and HL groups. Using the method described in [37], we calculated maximum steepness of the audiogram for the TIN and HL groups. There were no statistically significant differences in maximum steepness (p = 0.19 using Wilcoxon Rank Sum test) between the two groups. The hearing loss of all groups is depicted in Figure 1.

### Stimuli and Tasks

Stimuli used in the study consisted of pure tones and frequency modulated sweeps. There were three pure tones: 3 low frequency tones (0.5, 0.6, 0.7 kHz) and 3 high frequency tones (1.5, 1.7, 1.9 kHz). There were two types of frequency modulated sweep stimuli: “down-up” and “up-down”. The up-down stimuli consisted of a 200 ms up sweep, a 100 ms pure tone and a 200 ms down sweep. The three segments were concatenated such that each complete stimulus was continuous and the total duration was 500 ms. For both the up-down and the down-up stimuli, the 100 ms pure tone frequency was 1 kHz. There were 3 up-down stimuli with varying frequencies. The starting frequencies of the up-down stimuli were 0.65, 0.55, 0.45 kHz. The initial 200 ms up sweep always ended at 1 kHz (the frequency of the pure tone) regardless of the starting frequency. The down sweep then started at a frequency of 1 kHz and dropped in frequency to 0.65, 0.55 and 0.45 kHz to match each beginning frequency. The down-up stimuli were identical to the up-down in duration and segmentation but consisted of a concatenated down sweep, pure tone (with frequency at 1 kHz) and up sweep. There were 3 down-up stimuli with starting and ending frequencies of 2.0, 1.8, 1.6 kHz. Each stimulus-pair (either ‘same’ or ‘different’) was presented 5 times in pseudo-random order with 10 silent (rest) trials mixed amongst the listening trials. Thus, there were 30 trials with pure tones and 30 trials with the sweeps, with equal distribution of same and different trials. The tones and sweeps were generated using Audition 2.0 (Adobe Systems Inc., San Jose, CA). None of the stimuli overlapped the hearing loss range of the listeners who had normal hearing at frequencies less than 2 kHz. The sounds were further low-pass filtered with a cut-off point at 2 kHz and were normalized to have the same root-mean-square amplitude. Sounds were played at most comfortable level for the participants, during the ‘silent’ portion of the sparse sampling acquisition. Post-hoc measurements revealed that this was between 70–80 dB SPL.

Subjects performed (a) a passive listening task (PL) where they listened to pairs of stimuli without responding and (b) a discrimination task (DT) in which they responded whether a pair of tones or a pair of sweeps was ‘same’ or ‘different’. Responses were collected via button-presses. Subjects performed a brief training session for 5–10 minutes to familiarize them with the tasks and stimuli. The training sounds were similar to but not identical to the stimuli used in the experiment. Subjects began the actual experiment once they achieved a threshold of 85% accuracy on the task.

### Data Acquisition

**Figure 2:**
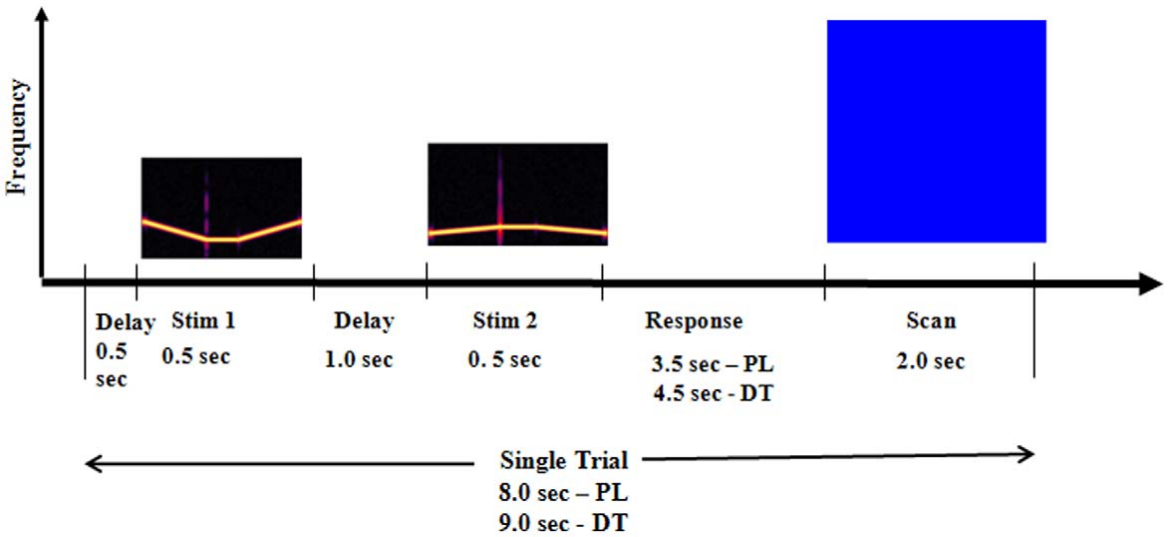
Timeline of a trial using sparse imaging technique. PL = passive listening, DT = discrimination task.

Participants were scanned in a 3 Tesla GE Excite scanner using an eight-channel receive-only coil. Subjects were scanned using an Echo Planar Imaging (EPI) sparse sampling technique (shown in Figure 2) so that the stimuli were presented in silence. Three different types of stimuli were used: pure tones, frequency modulated sweeps and music samples. Thirty-two T2*-weighted axial slices (TR = 2000 ms, TE = 30 ms) were collected for each volume in an interleaved order with a 2.6 mm slice thickness, 1.2 mm slice gap, and a 2.5 mm by 2.5 mm within plane resolution (96 by 96 Matrix, 240 mm FOV). We obtained 70 image volumes for each EPI run, including 60 image volumes for the task and 10 for the resting condition. Because of the timing of sparse sampling and BOLD delay, the image volume targeted neural activity related to the delay, the second stimulus and beginning of the response period. Subject behavior was recorded using button presses.

### Data Processing

Statistical parametric software (SPM5, Wellcome Trust Centre for Neuroimaging, http://www.fil.ion.ucl.ac.uk/spm/software/spm5/) was used to analyze the data. The fMRI data were pre-processed: images were realigned, co-registered to a high resolution template, normalized to MNI space, and finally smoothed with an 8-mm full-width-half-maximum filter. The smoothed data from individual subjects was entered into a fixed-effects analysis for purposes of statistical analyses at group level. A design matrix of all three groups was defined comprising contrasts testing for significant effects of various task components from each group. The contrasts were Task (either DT or PL)>Rest or Rest>Task. Because the PL and DT tasks were in separate EPI runs, they were analyzed separately. To analyze commonalities across the two hearing loss groups (TIN, HL), we performed a conjunction analysis [38]. Voxel clusters were considered to be statistically significant if they were p<0.05 corrected for multiple comparisons using family-wise error (FWE) either at the voxel or cluster-level, unless otherwise stated.

## Results

### Behavioral Results

There were no statistically significant differences between the three groups for the discrimination task, either in accuracy or response times. Behavioral responses of two normal hearing participants were excluded in the analysis: the button responses of one were inadvertently not recorded for all trials and the other only performed at 55% accuracy. We had set the inclusion criterion at 75% accuracy. All included participants, regardless of group, performed at or near ceiling; the lowest individual score was 87% accuracy. The group scores were as follows: normal hearing (N = 9, mean = 92, standard deviation = 5.75), hearing loss (N = 7, mean = 91.8, standard deviation = 4.65), tinnitus (N = 8, mean = 91.0, standard deviation = 4.25).

### fMRI Results

#### Passive Listening task

**Figure 3:**
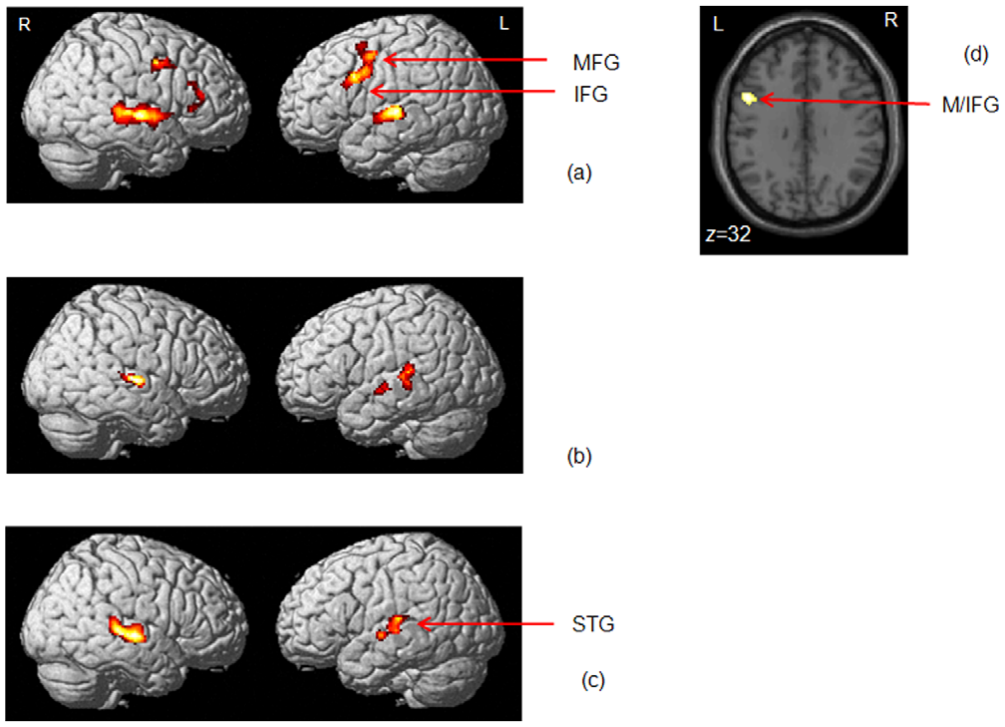
Statistical parametric maps of the passive listening task. Statistical parametric maps of the passive listening task (PL>Rest) rendered on a template brain for (a) normal hearing, (b) hearing loss and (c) tinnitus with hearing loss groups. Results of the NH>TIN comparison showed greater response in the left middle/inferior frontal gyri are depicted in (d). All reported clusters are p<0.05 FWE corrected for multiple comparisons at the voxel or cluster-level. Some clusters are highlighted in the figure - MFG: middle frontal gyrus, IFG: inferior frontal gyrus, STG: superior temporal gyrus.

As shown in Figure 3 and Table 2, all three groups, on average, showed greater response of the bilateral superior temporal cortex, including regions in the superior and middle temporal gyri and superior temporal sulcus, when listening to the stimuli compared to rest. We also observed greater response of some loci in bilateral inferior and middle frontal gyri for the normal hearing control group. When we performed pair-wise comparisons of the groups, the only statistically significant region of difference was in the left inferior and middle frontal gyri for the NH>TIN contrast (Figure 3D).

**Table 2:** Local maxima for the individual groups and the inter-group contrasts for the passive listening task compared to rest.

Contrast	MNI coordinates			Z score	Clustersize	Gyrus(Brodmann Area)
	*x*	*y*	*z*			
NH groupPL>Rest	−50	−20	2	6.09 ^*#^	597	L superior temporal gyrus (21/22)
	66	−16	−2	5.69^*#^	888	R superior and middle temporal gyri, superior temporal sulcus (21/22)
	−50	10	32	4.87^*#^	634	L inferior and middle frontal gyri (44/6)
	40	26	4	4.08^*^	150	R inferior and middle frontal gyri (45/46)
	44	0	40	3.84^*^	173	R middle frontal gyrus (9)
HL groupPL>Rest	−46	−32	12	5.62^*#^	455	L transverse and superior temporal gyri, superior temporal sulcus (41/42/22)
	46	−20	2	4.41^*^	293	R superior temporal gyrus (22)
TIN groupPL>Rest	60	−28	2	5.13^*#^	623	R superior and middle temporal gyri, superior temporal sulcus (42/22/21)
	−52	−12	−2	4.23^*^	358	L superior and middle temporal gyrus, superior temporal sulcus (42/22/21)
NH>TIN	−50	10	32	4.17^*^	300	L inferior and middle frontal gyri (44,6,9)

All reported clusters are p<0.05 FWE corrected for multiple comparisons at the voxel (indicated by ^*^ next to the Z-score) or cluster-level (indicated by ^#^), cluster extent is 50 voxels.

#### Discrimination task

**Figure 4:**
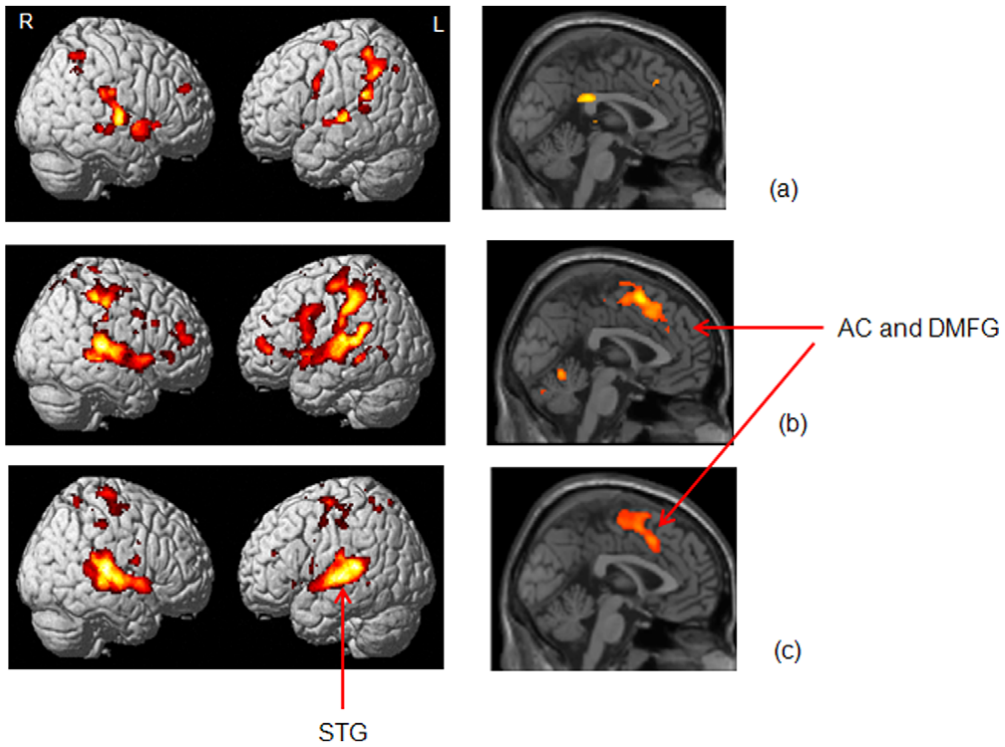
Statistical parametric maps of the discrimination task. Statistical parametric maps of the discrimination task (DT>Rest) rendered on a template brain for (a) normal hearing, (b) hearing loss and (c) tinnitus with hearing loss groups are shown on the left. The sagittal sections shown are located at *x* = 0 for all groups are shown on the right. All reported clusters are p<0.05 FWE corrected for multiple comparisons at the voxel or cluster-level. Some clusters are highlighted in the figure - AC: anterior cingulate, DMFG: dorsomedial frontal gyrus, STG: superior temporal gyrus.

All three groups, on average, showed greater response of the bilateral superior and middle temporal cortex when discriminating sounds compared to rest (see Figure 4 and Table 3). In addition, there was widespread activation of other regions that varied with subject group. Recall that all participants, regardless of the group, could hear the sounds and task performance across the groups was similarly at or near ceiling. On average, the normal hearing group activated the bilateral middle frontal gyrus, inferior parietal lobule, thalamus and putamen, central posterior cingulate, left cerebellum, and hippocampus and right superior frontal gyrus and anterior cingulate to a greater extent for the discrimination task relative to test. The HL group preferentially activated most of the same regions as the NH group, but to a greater extent, and additionally activated the left postcentral gyrus, right cerebellum and right caudate. The TIN group, on average, did not show wide-spread response in the frontal and parietal cortices for the discrimination task compared to rest, although, they exhibited activations in the anterior cingulate and dorsomedial frontal gyrus and in the bilateral precentral and postcentral gyri. We also conducted Rest>DT comparisons. If the Rest>DT contrast yielded extensive clusters of activation, a likely interpretation would be that there is higher activity in the rest condition rather than the task conditionfor those regions. Melcher et al. [9] have noted greater activity in the inferior colliculus in participants with tinnitus relative to those without tinnitus, suggesting increased baseline activity due to tinnitus. However, except for one suprathreshold cluster for the HL group in the left parahippocampal gyrus (MNI coordinates: −28, −44, −12), we did not find any activation clusters for the other groups for Rest>DT contrast. To investigate the effect of hearing loss and tinnitus we performed group-wise comparisons, the results of which are described next.

**Table 3:** Local maxima for the individual groups (cluster extent = 50 voxels) for the discrimination task compared to rest.

Contrast	MNI coordinates			Z score	Clustersize	Gyrus (Brodmann Area)
	*x*	*y*	*z*			
NH groupDT>Rest	58	−12	2	Inf^*#^	681	R superior and middle temporal gyrus (22,21)
	−44	−42	24	8.62^*#^	898	L transverse and superior temporal gyrus, inferior parietal lobule (42,22,40)
	54	4	−8	7.6^*#^	249	R superior and middle temporal gyrus (22,21)
	−40	−20	−12	6.5^*#^	112	L hippocampus
	12	−10	10	6.44^*#^	528	R thalamus, putamen
	−52	2	30	6.39^*#^	123	L inferior and middle frontal gyrus (44, 6)
	−52	−20	2	6.34^*#^	274	L superior and middle temporal gyrus (22,21)
	36	−48	38	6.25^*#^	128	L inferior parietal lobule (40)
	0	−34	26	6.16^*#^	110	posterior cingulate (23)
	−34	−10	62	6.12^*#^	86	L precentral gyrus, middle frontal gyrus (4,6)
	−26	−66	−36	5.99^*#^	51	L cerebellum
	−22	14	−2	5.82^*#^	88	L putamen
	48	−54	52	5.79^*#^	92	R inferior parietal lobule (40)
	8	26	40	5.46^*#^	54	R anterior cingulate, dorsomedial frontal gyrus (32, 8)
	36	44	26	5.32^*#^	60	R middle and superior frontal gyrus (10)
	44	−24	58	5.23^*#^	36	L thalamus, putamen
HL groupDT>Rest	60	−28	8	Inf^*#^	2804	R superior temporal gyrus (42, 22)
	−54	−44	22	Inf^*#^	4318	L postcentral gyrus, inferior parietal lobule (1, 2, 3, 40)
	0	10	58	Inf	1039	Dorsomedial frontal gyrus, anterior cingulate (6, 8, 32)
	−36	50	10	6.89^*#^	232	L middle frontal gyrus (10,44)
	−54	−40	−4	6.5^*#^	129	L superior and middle temporal gyrus (22, 21)
	2	−62	−10	6.5^*#^	98	R cerebellum
	34	52	14	6.49^*#^	345	R middle and inferior frontal gyrus (10, 46)
	8	−76	−24	6.15^*#^	104	R cerebellum
	24	14	0	6.11^*#^	389	R putamen
	16	−2	10	6.11^*#^	129	R putamen, caudate
	−6	34	28	5.97^*#^	54	L anterior cingulate, dorsomedial frontal gyrus (32, 9)
	−18	−58	−32	5.91^*#^	65	L cerebellum
	54	4	36	5.87^*#^	56	R inferior frontal gyrus (44)
	−10	−20	4	5.86^*#^	76	L thalamus
	−30	−60	46	5.4^*#^	56	L inferior parietal lobule (40)
TIN groupDT>Rest	−58	−22	4	Inf^*#^	2372	L superior temporal gyrus (22)
	54	−26	10	Inf^*#^	2806	R superior temporal gyrus (42, 22)
	36	−22	70	Inf^*#^	260	R precentral gyrus, superior frontal gyrus (4, 6)
	−2	12	44	6.69^*#^	846	L dorsomedial frontal gyrus, anterior cingulate (6, 32)
	−22	6	6	6^*#^	356	L putamen
	−40	−14	64	5.97^*#^	133	Left precentral and postcentral gyri (4, 1, 2, 3)
	60	4	16	5.86^*#^	91	R inferior frontal gyrus (44, 6)
	34	−52	64	5.74^*#^	56	R postcentral gyrus, superior parietal lobule (5, 7)
	24	6	4	5.63^*#^	164	R putamen
	56	−30	44	5.55^*#^	57	R postcentral gyrus (1, 2, 3)
	−38	−58	56	5.38^*#^	56	L inferior and superior parietal lobule (40, 5, 7)

All reported clusters are p≤0.05 FWE corrected for multiple comparisons at the voxel (indicated by ^*^ next to the Z-score) or cluster-level (indicated by ^#^).

#### Effect of Hearing Loss

**Figure 5:**
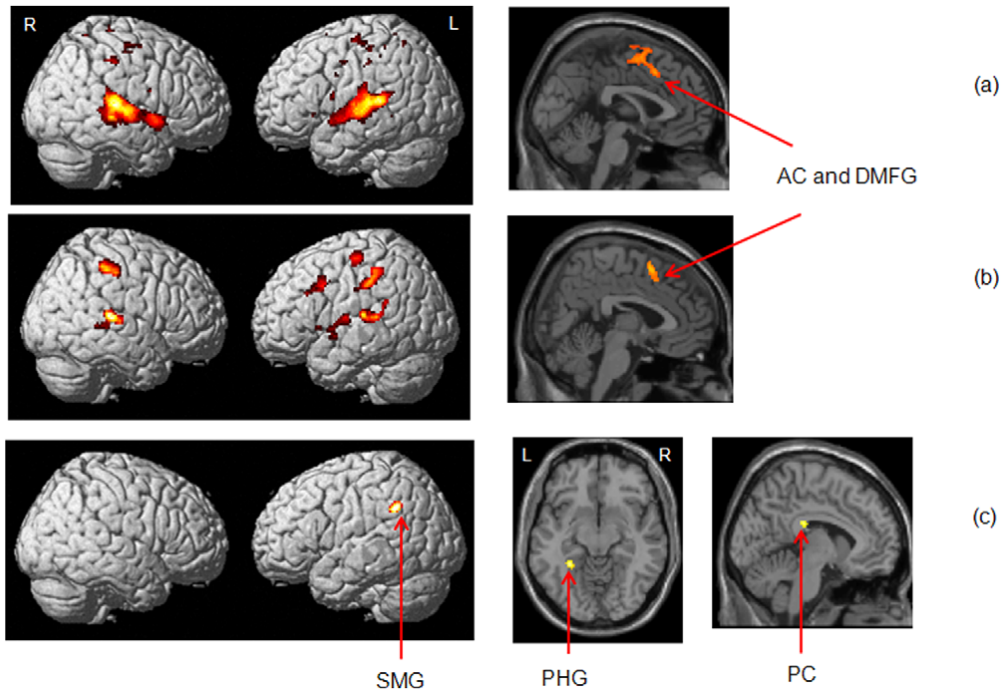
Effect of hearing loss. Statistical parametric maps for (a) conjunction of HL and TIN rendered on a template brain and sagittal slice at *x* = 0, (b) contrast HL>NH rendered on a template brain and sagittal slice at *x* = 4, and (c) contrast NH>HL rendered on a template brain, axial slice at *z* = −12, and sagittal slice at *x* = −6. All reported clusters are p<0.05 FWE corrected for multiple comparisons at the voxel or cluster-level. Some clusters are highlighted in the figure - AC: anterior cingulate, DMFG: dorsomedial frontal gyrus, SMG: supramarginal gyrus, PHG: parahippocampal gyrus, PC: posterior cingulate.

Conjunction analysis, which was used to identify commonalities between HL and TIN groups, revealed widespread activations in the bilateral superior temporal cortex and in the central regions of anterior cingulate and in the medial frontal gyri (Figure 5A, left and right, respectively).

We next determined the effect of hearing loss alone without the confounding factor of tinnitus, by comparing HL and NH groups (Table 4). The following regions showed increased response for the HL group compared to the NH control group during discrimination: bilateral superior temporal gyrus, right postcentral gyrus, right inferior parietal lobule, left precentral gyrus, left superior frontal gyrus and left transverse temporal gyrus (Figure 5B, left) and central dorsomedial frontal gyrus, central anterior cingulate (Figure 5B, right). For the reverse contrast of NH>HL, the suprathreshold voxel clusters were in the left posterior cingulate (Figure 5C, right), left inferior parietal lobule, including the supramarginal gyrus (Figure 5C, left) and the left parahippocampal gyrus (Figure 5C, center).

**Table 4:** Local maxima for the inter-group contrasts (cluster extent = 20 voxels) for the discrimination task compared to rest.

Contrast	MNI coordinates			Z score	Clustersize	Gyrus (Brodmann Area)
	*x*	*y*	*z*			
HL>NH	58	−26	48	6.37^*#^	203	R postcentral gyrus (1,2)
	60	−28	8	5.66^*#^	286	R superior temporal gyrus (42, 22)
	−56	−30	38	5.64^*#^	276	R postcentral gyrus, inferior parietal lobule (1, 2, 40)
	−46	−18	60	5.15^#^	106	L precentral gyrus (4)
	−44	−28	12	5.02^*#^	312	L transverse and superior temporal gyrus (42, 22)
	−32	−26	72	4.79^#^	29	L superior frontal gyrus (6)
	−50	−22	−4	4.75^#^	68	L superior and middle temporal gyri (21)
	−2	10	56	4.61^*#^	203	R dorsomedial frontal gyrus, anterior cingulate (8, 6,32)
NH>HL	−28	−44	−12	4.7^#^	54	L parahippocampal gyrus (37, 36)
	−4	−28	24	4.69^#^	113	L posterior cingulate (23)
	−52	−48	36	4.67#	86	L inferior parietal lobule, supramarginal gyrus (40)
HL>TIN	−54	−44	22	6.07^#^	88	L superior temporal gyrus (42, 22)
	−36	52	10	5.03^#^	163	L superior and middle frontal gyri (10)
	54	22	26	4.71^#^	73	R inferior frontal gyrus (44, 46)
	−50	−34	42	4.52^#^	152	L inferior parietal lobule (40)
	−50	10	36	4.2^*^	150	L inferior frontal gyrus (44)
	6	−78	−24	4.14^*^	182	R cerebellum
	−42	−68	4	4.09^*^	164	L middle temporal gyrus (22, 37)
TIN>HL	No significant differences					
TIN>NH	−50	−38	8	5.62^*#^	979	L transverse and superior temporal gyrus (41, 42, 22)
	54	−26	8	5.26^*#^	451	R superior and middle temporal gyrus (42, 22, 21)
	36	−22	70	5.04^#^	34	R superior frontal gyrus (6)
NH>TIN	No significant differences					

All reported clusters are p≤0.05 FWE corrected for multiple comparisons at the voxel (indicated by ^*^ next to the Z-score) or cluster-level (indicated by ^#^).

#### Effect of Tinnitus

**Figure 6:**
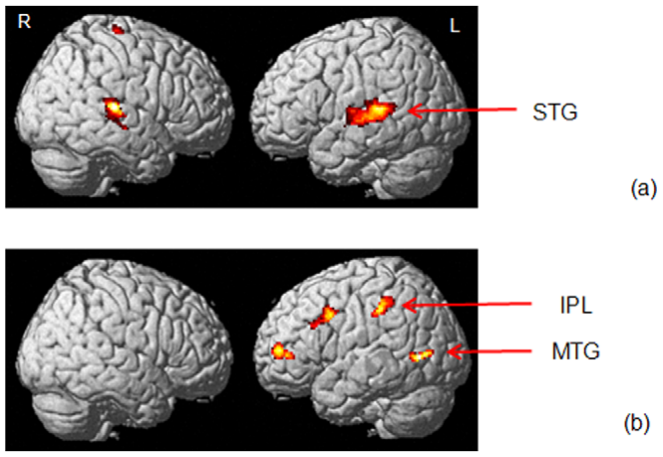
Effect of tinnitus. Statistical parametric maps rendered on a template brain for the contrasts (a) TIN>NH and (b) HL>TIN, showing increased and decreased response, respectively, due to tinnitus. The contrasts NH>TIN and TIN>HL did not result in any suprathreshold voxels. All reported clusters are p<0.05 FWE corrected for multiple comparisons at the voxel or cluster-level. Some clusters are highlighted in the figure – STG: superior temporal gyrus, IPL: inferior parietal lobule, MTG: middle temporal gyrus.

We contrasted the activation patterns for the TIN group separately against the NH and HL groups (Figure 6, Table 4). We observed increased response for the TIN group relative to the NH group in left transverse and superior temporal gyri, right superior frontal gyrus, and right superior and middle temporal gyri (Figure 6A). We did not find any suprathreshold clusters of voxels for the TIN>HL contrast. We observed an extensive network of regions showing decreased response for the TIN group in the HL>TIN contrast in the left superior and middle temporal gyri, bilateral inferior frontal gyri, left inferior parietal lobule, left superior and middle frontal gyri and right cerebellum (Figure 6B). We did not find any suprathreshold clusters of voxels for the NH>TIN contrast, although there was a trend (p = 0.16 FWE corrected) at right superior frontal gyrus.

## Discussion

Our study employed passive listening and active discrimination tasks to investigate differences in the neural bases of hearing loss and chronic tinnitus. We found bilateral superior temporal cortex response for passive listening of sounds across all three groups (hearing loss, hearing loss with tinnitus, and normal hearing without tinnitus). There were no regions of significant difference for the passive listening task between the three groups, except participants in the NH group activated the left inferior/middle frontal gyrus to a greater extent relative to those in the hearing loss groups with and without tinnitus (TIN and HL). The patterns of response across the three groups varied more for the discrimination task compared to the passive listening task. In the discrimination task compared to rest, we found an elevated response in the frontal and parietal cortices in addition to the temporal cortex for the normal hearing and hearing impaired without tinnitus participants. This is not surprising because the discrimination task is a short-term memory task. The activation patterns seen in the normal hearing control group are similar to those seen in our previous study of young normal hearing adults [29]. However, we observed an increased response of the frontal and parietal cortices for the HL group relative to the NH and TIN groups. The TIN group demonstrated heightened response in bilateral temporal and left frontal cortices when compared with the NH group and decreased response in the temporal, frontal and parietal cortices with respect to the HL group.

One of the surprising results of our study was that high-frequency hearing loss affected perception and discrimination of low-frequency sounds, not in terms of behavior, but in terms of the response of the auditory, frontal and parietal cortices. Because the sounds were low-pass filtered at 2 kHz to be within the normal hearing range of all participants, the null hypothesis was that there would be no difference in the response of brain for the participants regardless of their hearing status (disregarding tinnitus). This was mostly true for the passive listening task; however, we observed differential involvement of a distributed set of brain regions in the three groups for the discrimination task, varying both on hearing and tinnitus status.

The distributed set of regions (prefrontal cortex, inferior parietal cortex, anterior cingular cortex) highlighted in our results have been proposed previously to play an important role in auditory attention and working memory. Although our study did not employ tasks that require attention explicitly, the short-term memory task uses attention implicitly. Studies investigating attention in the auditory modality have reported on the involvement of the following cortical and subcortical structures: prefrontal cortex, parietal cortex, superior temporal gyrus, temporoparietal junction, anterior cingulate gyrus, basal ganglia, thalamus and inferior colliculus (for reviews, see [39], [40], [41], [42], [43]). Whereas the putative attention and working memory network can be large and distributed [43], [44], for the purposes of the present study and to simplify terminology, we will use the term auditory attention and short-term memory (AASM) network and restrict its definition to include frontal cortex, parietal cortex, superior temporal gyrus and anterior cingulate cortex.

Cognitive scientists and neuroscientists have suggested that cognitive function may be affected by sensory difficulties in older adults [45]. The incoming signal, the receiver (ear, cochlea) and the perception/cognition system may all contribute to imperfect cognitive performance. Several cognitive theories have been forwarded that relate the interaction of these agencies. One such theory, the ‘effortfulness hypothesis’ [46], holds that either with degraded sensory abilities [47] or in noisy conditions [48] individuals tend to increase effort to achieve successful perception. Increased effort in initial stages of processing drains cognitive resources from speech processing and other higher-level cognitive processes [45], [49], [50]. Treisman's ‘levels of analysis’ model of attention [51], [52] provides a framework for investigating bottom-up auditory and top-down cognitive interactions [53] due to both hearing loss and tinnitus. Using such a framework to study hearing in adults allows us to understand the dynamic, competitive, and ‘mutually compensatory’ activity [53] between auditory and cognitive factors, and where attentional resources may be allocated. Attentional resources apply to working memory [54] by limiting processing of unattended inputs and facilitating that of attended inputs [55] and may be used to index effort due to hearing loss or tinnitus.

We interpret the differential response of the AASM network as follows. The hearing loss group likely engages attentional resources to a greater extent compared to normal hearing participants in order to compensate for their hearing impairment. The hearing loss group activates superior temporal, superior frontal, inferior parietal, and anterior cingular cortices significantly more than the normal hearing group (Table 4). Note that normal hearing participants exhibited marginal involvement of the anterior cingulate (just above threshold) and they appeared also to engage the frontal and parietal cortices (Table 3). Further, our results suggest that those who, in addition to having hearing loss, perceive tinnitus use their attentional resources in a different manner. We speculate that attention needs to be diverted to a phantom sound while at the same time actively processing external, relevant stimuli. In order to attend to external stimuli, subjects with tinnitus (similar to other groups) activated superior and middle temporal cortex and anterior cingulate to a greater extent for the discrimination task when contrasted to rest. However, compared to the hearing loss group, there was less widespread response of the superior and middle frontal gyri and inferior parietal cortices by the tinnitus group for the discrimination task relative to rest (Table 4). This implies that the compensatory mechanisms differ for tinnitus and hearing loss and may result in differing functional neural response. Nevertheless, studies explicitly targeting the attentional network in the two hearing loss groups, with and without tinnitus, are needed to confirm the role of the AASM in hearing loss and chronic tinnitus.

The role of the attentional network in mediating chronic tinnitus has been inferred from a number of whole-brain imaging studies. Use of lidocaine allowed [13] to temporarily suppress tinnitus in their participants; subtracting the tinnitus-suppressed blood flow pattern from that during the tinnitus perception state led to the visualization of a broad temporal-parietal network possibly related to attention and a paralimbic network possibly related to emotion. By employing aversive stimuli to simulate tinnitus-like conditions in normal hearing volunteers without tinnitus, [56] also found dorsolateral prefrontal and paralimbic structures to be responsive to the aversive sounds. Mirz et al. contrasted tinnitus perception with tinnitus suppression to identify a frontal-temporal network as associated with chronic tinnitus sensation [11]. All these studies found auditory and attention processing regions in the cortex to be involved in mediating tinnitus perception. However, few imaging studies have investigated involvement of the attentional network using behavioral tasks.

Neurophysiological studies employing electroencephalography (EEG) have also implicated the involvement of the attentional network in tinnitus perception; however, for the most part they have not taken into account the effect of hearing loss and have reported disparate findings. Jacobson and colleagues [6] observed that an electrophysiological index of early selective auditory attention (‘negative difference wave’) was greater and the N100 component occurred later in tinnitus patients relative to controls, suggesting there are differences in early selective attention between patients with bothersome tinnitus and controls. The negative difference wave was obtained by subtracting the event-related potential (ERP) component of an ignore-frequent-stimulus from the ERP component of an attend-frequent-stimulus. The participants in the study had high-frequency hearing loss (not affecting thresholds at 0.5 and 1 kHz) whereas the controls had normal hearing. A later study by [57] however, did not find any group differences in latency of N100 across passive and selective listening conditions. In a recent event-related potentials study [58], high distress related to tinnitus was associated with smaller changes in the event-related potentials between attended and unattended auditory task conditions. Both controls and those with mild tinnitus exhibited greater changes in N100 amplitude and phase locking between the attended and unattended task conditions. Tinnitus patients may have had some high-frequency hearing loss; the criterion for inclusion was normal hearing up to 2 kHz. Although our results suggest differential involvement of the attentional network, the study was not explicitly designed to test attentional load. There remains a need to conduct a brain imaging study explicitly investigating the role of the attentional network in tinnitus perception and delineate it from the response of the attentional network in hearing loss alone.

Although we found brain activation pattern differences between the groups, we did not find any statistically significant behavioral differences. Behavioral studies have noted attentional deficits in selective and divided attention in chronic tinnitus sufferers, specifically in the form of slower response times. In one such study [25], investigators found slower reaction times in individuals with severe tinnitus relative to controls in visual Stroop tasks with color and word naming components and in a demanding dual task involving word reading or category naming. Dornhoffer et al. [23] found no significant differences between individuals with tinnitus and controls in terms of arousal to a repetitive sensory stimulation as measured by a brainstem-thalamus P50 potential or by habituation as measured by the ability to suppress a second stimulus. They did, however, find statistically significant slower reaction times in the tinnitus group compared to the control group. This is in contrast to our study, where we found cortical-level activation differences between tinnitus and non-tinnitus groups but no reaction-time differences at the behavioral level. Hallam et al. [24] have posited that tinnitus impairs cognitive efficiency because of the self-reported concentration problems by tinnitus sufferers. In their study, Hallam et al. [24] tested tinnitus and non-tinnitus sufferers on five cognitive tasks that probed sustained attention, reaction time, verbal fluency and immediate and delayed memory. The tinnitus group was slower than the hearing-impaired and normal hearing control groups in reaction time tasks, but had similar behavior on the verbal fluency task as the hearing impaired group, and both groups performed worse than the normal hearing group. The other tasks showed similar performances between the groups. Hallam et al. interpreted these results as suggesting that ‘cognitive inefficiency in tinnitus participants is related to the control of attentional processes’ (page 218, [24]). With regards to the present study, it is possible that the discrimination task used did not engage the attentional system to the extent that resulted in differences in behavior. Nevertheless, even without overt behavioral differences our results showed that there were differences in the attentional network among the three groups for the same task.

We compared TIN and HL groups in order to understand better the neural correlates of tinnitus, however, the interaction between hearing loss and tinnitus may not be linear. The TIN and HL comparison gave us greater understanding of the brain regions most affected by hearing loss and those that may be most influenced by tinnitus, within the context of simple listening tasks. The relation between hearing loss and tinnitus is complex and includes other cortical and subcortical networks such as those subserving emotion or somatosensory processing [14], [17]. Whereas peripheral hearing loss may be the most prominent trigger for tinnitus, pathophysiology resulting in tinnitus may be quite different than in hearing loss without tinnitus [59], [60]. Further, the compensatory effects in the two conditions (hearing loss with tinnitus and hearing loss without tinnitus) may be different and not necessarily become evident by direct comparisons. In order to control for some aspects of hearing loss, we carefully matched the hearing loss profiles for the HL and TIN groups. However, it is possible that other peripheral hearing factors such as cochlear dead regions, differences in hearing loss slopes, differences in etiology of hearing loss between the two groups may be responsible for some of the variations seen in the fMRI results. As is true of almost any patient population and particularly so of tinnitus, the tinnitus patient population is heterogeneous [4]. The type of tinnitus, laterality, duration of tinnitus, pitch and loudness of the percept, presence of hyperacusis, concomitant disorders such as depression and anxiety may influence the neural correlates of tinnitus.

We collected structural MR and diffusion tensor imaging (DTI) data on the same group of participants in the current study [27]. We observed that the hearing loss group without tinnitus had the most profound changes in both white and gray matter relative to the other groups, those with normal hearing and those with hearing loss accompanied by tinnitus. This is in contrast to our fMRI findings that showed large scale changes in both the tinnitus and hearing loss without tinnitus groups. The gray matter decreases seen in the HL group relative to TIN and NH groups were in the anterior cingulate, putamen and the middle frontal gyrus. These changes are in the same regions as the reductions seen in the hearing loss group and the tinnitus group for the discrimination task compared to rest contrast in the fMRI study. Impaired sustained attention may have an impact on gray matter, as has been observed in studies of schizophrenia [61]. This suggests that functional compensation due to sensory deprivation may result in long-term structural changes. There was no significant alteration of the gray or white matter in the TIN group compared to the NH group.

We chose to perform a fixed-effects analysis because of the limited number of participants in our study and additionally, the patients belonged to a subset (bilateral hearing loss with mild tinnitus) of a complex heterogeneous population. Fixed-effects analysis also lends itself to conjunction analysis [38] as we have demonstrated in Figure 5. Conjunction analysis allows us to identify common activation patterns across groups. In the study, we used conjunction analysis to characterize shared neural correlates of the two hearing loss groups. Regardless of the analytical method, generalization of the study results to the larger patient group of those with tinnitus is limited beyond a systems-level interpretation. For instance, individuals with unilateral tinnitus and normal hearing may exhibit different responses compared to the patients in the present study, at a lower sensory or subcortical level, although they may exhibit commonalities at a higher, non-sensory level. It is also likely that those with severe tinnitus may exhibit different responses of the AASM network compared to the participants with mild tinnitus in our study. Meta-analysis of a series of brain imaging studies employing short-term working memory tasks with differing attentional demands in individuals with different types of tinnitus may allow us to draw more robust conclusions about the role of the attentional network in hearing loss or tinnitus.

In conclusion, our study suggests the differential involvement of a putative AASM network in hearing loss with and without tinnitus. This network consisted of regions in the frontal, parietal and temporal cortices and the anterior cingulate. In participants with hearing loss without tinnitus, the attentional network response was enhanced relative to normal hearing controls. In individuals with tinnitus and hearing loss, the response of some nodes of the attentional network was diminished with respect to hearing loss only group, whereas response of other nodes was enhanced. This suggests a complex role for the attentional network in those with chronic tinnitus and studies are needed that will elaborate on the functioning of this network.

## References

[1] Davis A, Rafaie EA, Tyler RS (2000). Epidemiology of tinnitus.. Tinnitus Handbook.

[2] Lockwood AH, Salvi RJ, Burkard RF (2002). Tinnitus.. N Engl J Med.

[3] Vernon JA (1997). (1997) Tinnitus: treatment and relief.

[4] Moller AR (2007). Tinnitus: presence and future.. Prog Brain Res.

[5] Sindhusake D, Golding M, Newall P, Rubin G, Jakobsen K (2003). Risk factors for tinnitus in a population of older adults: the blue mountains hearing study.. Ear Hear.

[6] Jacobson GP, Calder JA, Newman CW, Peterson EL, Wharton JA (1996). Electrophysiological indices of selective auditory attention in subjects with and without tinnitus.. Hear Res.

[7] Landgrebe M, Langguth B, Rosengarth K, Braun S, Koch A (2009). Structural brain changes in tinnitus: grey matter decrease in auditory and non-auditory brain areas.. Neuroimage.

[8] Muhlau M, Rauschecker JP, Oestreicher E, Gaser C, Rottinger M (2006). Structural brain changes in tinnitus.. Cereb Cortex.

[9] Melcher JR, Sigalovsky IS, Guinan JJ, Levine RA (2000). Lateralized tinnitus studied with functional magnetic resonance imaging: abnormal inferior colliculus activation.. J Neurophysiol.

[10] Lockwood AH, Salvi RJ, Coad ML, Towsley ML, Wack DS (1998). The functional neuroanatomy of tinnitus: evidence for limbic system links and neural plasticity.. Neurology.

[11] Mirz F, Gjedde A, Ishizu K, Pedersen CB (2000). Cortical networks subserving the perception of tinnitus–a PET study.. Acta Otolaryngol Suppl.

[12] Mirz F, Pedersen B, Ishizu K, Johannsen P, Ovesen T (1999). Positron emission tomography of cortical centers of tinnitus.. Hear Res.

[13] Andersson G, Lyttkens L, Hirvela C, Furmark T, Tillfors M (2000). Regional cerebral blood flow during tinnitus: a PET case study with lidocaine and auditory stimulation.. Acta Otolaryngol.

[14] Cacace AT (2003). Expanding the biological basis of tinnitus: crossmodal origins and the role of neuroplasticity.. Hear Res.

[15] Giraud AL, Chery-Croze S, Fischer G, Fischer C, Vighetto A (1999). A selective imaging of tinnitus.. Neuroreport.

[16] Kaltenbach JA (2006). The dorsal cochlear nucleus as a participant in the auditory, attentional and emotional components of tinnitus.. Hear Res.

[17] Bauer CA (2004). Mechanisms of tinnitus generation.. Curr Opin Otolaryngol Head Neck Surg.

[18] Tun PA, McCoy S, Wingfield A (2009). Aging, hearing acuity, and the attentional costs of effortful listening.. Psychol Aging.

[19] Parbery-Clark A, Strait DL, Anderson S, Hittner E, Kraus N (2011). Musical experience and the aging auditory system: implications for cognitive abilities and hearing speech in noise.. PLoS One.

[20] Stewart R, Wingfield A (2009). Hearing loss and cognitive effort in older adults' report accuracy for verbal materials.. J Am Acad Audiol.

[21] Craik FI, Byrd M, Craik FI, Trehub S (1982). Aging and cognitive deficits: the role of attentional resources.. Aging and Cognitive Processes.

[22] Salthouse TA (1994). The aging of working memory.. Neuropsychology.

[23] Dornhoffer J, Danner C, Mennemeier M, Blake D, Garcia-Rill E (2006). Arousal and attention deficits in patients with tinnitus.. Int Tinnitus J.

[24] Hallam RS, McKenna L, Shurlock L (2004). Tinnitus impairs cognitive efficiency.. Int J Audiol.

[25] Stevens C, Walker G, Boyer M, Gallagher M (2007). Severe tinnitus and its effect on selective and divided attention.. Int J Audiol.

[26] Brown SC, Johnson RC, Hotto SA (1990). Older Americans and tinnitus: A demographic study and chartbook.. GRI monograph series A, No 2: Gallaudet Research Institute, Gallaudet University.

[27] Husain FT, Medina RE, Davis CW, Szymko-Bennett Y, Simonyan K (2011). Neuroanatomical changes due to hearing loss and chronic tinnitus: a combined VBM and DTI study.. Brain Res.

[28] Wong PC, Ettlinger M, Sheppard JP, Gunasekera GM, Dhar S (2010). Neuroanatomical characteristics and speech perception in noise in older adults.. Ear Hear.

[29] Husain FT, Fromm SJ, Pursley RH, Hosey LA, Braun AR (2006). Neural bases of categorization of simple speech and nonspeech sounds.. Hum Brain Mapp.

[30] Wunderlich AP, Schonfeldt-Lecuona C, Wolf RC, Dorn K, Bachor E (2009). Cortical Activation during a Pitch Discrimination Task in Tinnitus Patients and Controls - An fMRI Study.. Audiol Neurootol.

[31] McCombe A, Baguley D, Coles R, McKenna L, McKinney C (2001). Guidelines for the grading of tinnitus severity: the results of a working group commissioned by the British Association of Otolaryngologists, Head and Neck Surgeons, 1999.. Clin Otolaryngol Allied Sci.

[32] Newman CW, Jacobson GP, Spitzer JB (1996). Development of the Tinnitus Handicap Inventory.. Arch Otolaryngol Head Neck Surg.

[33] Beck AT, Steer RA (1984). Internal consistencies of the original and revised Beck Depression Inventory.. J Clin Psychol.

[34] Steer RA, Clark DA, Beck AT, Ranieri WF (1999). Common and specific dimensions of self-reported anxiety and depression: the BDI-II versus the BDI-IA.. Behav Res Ther.

[35] Gu JW, Halpin CF, Nam EC, Levine RA, Melcher JR (2010). Tinnitus, diminished sound-level tolerance, and elevated auditory activity in humans with clinically normal hearing sensitivity.. J Neurophysiol.

[36] Clark JG (1981). Uses and abuses of hearing loss classification.. ASHA.

[37] Konig O, Schaette R, Kempter R, Gross M (2006). Course of hearing loss and occurrence of tinnitus.. Hear Res.

[38] Friston KJ, Holmes AP, Price CJ, Buchel C, Worsley KJ (1999). Multisubject fMRI studies and conjunction analyses.. Neuroimage.

[39] Hugdahl K, Westerhausen R, Alho K, Medvedev S, Laine M (2009). Attention and cognitive control: unfolding the dichotic listening story.. Scand J Psychol.

[40] Janata P (2005). Brain networks that track musical structure.. Ann N Y Acad Sci.

[41] Palmer AR, Hall DA, Sumner C, Barrett DJ, Jones S (2007). Some investigations into non-passive listening.. Hear Res.

[42] Shinn-Cunningham BG, Best V (2008). Selective attention in normal and impaired hearing.. Trends Amplif.

[43] Fritz JB, Elhilali M, David SV, Shamma SA (2007). Auditory attention–focusing the searchlight on sound.. Curr Opin Neurobiol.

[44] Deco G, Corbetta M (2011). The Dynamical Balance of the Brain at Rest.. Neuroscientist.

[45] Lindenberger U, Baltes PB (1994). Sensory functioning and intelligence in old age: a strong connection.. Psychol Aging.

[46] Rabbitt PM (1968). Channel-capacity, intelligibility and immediate memory.. Q J Exp Psychol.

[47] McCoy SL, Tun PA, Cox LC, Colangelo M, Stewart RA (2005). Hearing loss and perceptual effort: downstream effects on older adults' memory for speech.. Q J Exp Psychol A.

[48] Gao X, Stine-Morrow EA, Noh SR, Eskew RT (2010). Visual noise disrupts conceptual integration in reading.. Psychon Bull Rev.

[49] Li KZ, Lindenberger U (2002). Relations between aging sensory/sensorimotor and cognitive functions.. Neurosci Biobehav Rev.

[50] Pichora-Fuller MK, Souza PE (2003). Effects of aging on auditory processing of speech.. Int J Audiol.

[51] Treisman AM (1964). Selective Attention in Man.. Br Med Bull.

[52] Treisman AM (1969). Strategies and models of selective attention.. Psychol Rev.

[53] Craik FI (2007). The role of cognition in age-related hearing loss.. J Am Acad Audiol.

[54] Baddeley A (1992). Working memory.. Science.

[55] Sussman ES, Bregman AS, Wang WJ, Khan FJ (2005). Attentional modulation of electrophysiological activity in auditory cortex for unattended sounds within multistream auditory environments.. Cogn Affect Behav Neurosci.

[56] Mirz F, Gjedde A, Sodkilde-Jrgensen H, Pedersen CB (2000). Functional brain imaging of tinnitus-like perception induced by aversive auditory stimuli.. Neuroreport.

[57] Jacobson GP, McCaslin DL (2003). A reexamination of the long latency N1 response in patients with tinnitus.. J Am Acad Audiol.

[58] Delb W, Strauss DJ, Low YF, Seidler H, Rheinschmitt A (2008). Alterations in Event Related Potentials (ERP) associated with tinnitus distress and attention.. Appl Psychophysiol Biofeedback.

[59] Moller AR (2003). Pathophysiology of tinnitus.. Otolaryngol Clin North Am.

[60] Syka J (2002). Plastic changes in the central auditory system after hearing loss, restoration of function, and during learning.. Physiol Rev.

[61] Salgado-Pineda P, Baeza I, Perez-Gomez M, Vendrell P, Junque C (2003). Sustained attention impairment correlates to gray matter decreases in first episode neuroleptic-naive schizophrenic patients.. Neuroimage.

